# The MINOUWApp: a web-based tool in support of by-catch and discards management

**DOI:** 10.1007/s10661-020-08704-5

**Published:** 2020-11-09

**Authors:** Lorenzo D’Andrea, Aida Campos, Karim Erzini, Paulo Fonseca, Simone Franceschini, Stefanos Kavadas, Irida Maina, Francesc Maynou, Tommaso Russo

**Affiliations:** 1 Università degli studi di Roma Tor Vergata (UTV), Via della Ricerca Scientifica 1, 00133 Rome, Italy; 2 Centre of Marine Sciences (CCMAR), Universidade do Algarve, Campus de Gambelas, 8005-139 Faro, Portugal; 3 Instituto Português do Mar e da Atmosfera (IPMA), Avenida Alfredo Magalhães Ramalho 6, 1495-165 Algés, Portugal; 4 Hellenic Centre for Marine Research (HCMR), Institute of Marine Biological Resources and Inland Waters, 46.7 km Athens Sounio ave. P.O. Box 712, 19013 Anavyssos/Attiki, Greece; 5 Institut de Ciències del Mar (CSIC), Psg Marítim de la Barceloneta, 37-49 08003 Barcelona, Spain

**Keywords:** Fishing footprint;, Resource distribution;, Interactive application;, Participative management;, Landing obligation;, Decision support

## Abstract

Current fishing practices often do not allow adequate selection of species or sizes of fish, resulting in unwanted catches, subsequently discarded, with the consequent negative effects on both marine communities and fisheries profitability. The cross-analysis of density patches of potential unwanted catches and distribution of fishing effort can support the identification of spatial-temporal hot-spots in which the fishing pressure should be reduced to limit the amount of discards. The MinouwApp represents a technological and methodological framework to bring different, and structurally complex, sources of georeferenced data together into a simple visual interface aiming to interactively explore temporal ranges and areas of interest. The objective is to improve the understanding of fisheries dynamics, including discards, thus contributing to the implementation of discard management plans in a context of participative, ecosystem-based fisheries management strategies.

## Introduction

Balancing commercial exploitation of marine resources and environmental protection represents one of the main global issues and probably the greatest challenge in fisheries science (Nelson and Burnside 2019). In a context of climate change, ecosystem degradation and population growth that contribute to impair the effectiveness of management strategies (Sumaila et al. 2011*;* Cheung et al. 2013), credible advice for policy-making depends on ensuring timely access to relevant data resulting from public-funded research, as recommended by the European Commission (2012) and the “European Code of Conduct for Research Integrity” (ALLEA 2017). The development of interactive applications storing spatial data about the distribution of human activities is essential to allow exploring their overlap and impact on marine ecosystem resources (Halpern et al. 2008). One of the main fisheries-related impacts is historically represented by the practice of discarding during fishing operations. Within the new Common Fisheries Policy (CFP—https://ec.europa.eu/fisheries/cfp_en), discarding is defined as “the practice of returning unwanted catches (UWC) to the sea, either dead or alive, because they are undersized, due to market demand, the fisherman has no quota or because catch composition rules impose this.” It is largely acknowledged that UWC and discards cause unacceptable damage to the marine communities since they represent an additional, usually unaccounted source of mortality for the stocks and a threat to the ecosystems. Discarding is a pervasive practice in fisheries, and it is estimated that globally, discards account for between 10 and 23% of total catches (Guillen et al. 2018*;* Pérez Roda et al. 2019). According to these concerns, one of the more recent initiatives of the European Community towards marine resource protection and fisheries optimization is represented by the strategy towards minimization of UWC through the implementation of a landing obligation (LO), i.e., the interdiction of discarding, for commercial species subject to output control measures such as the definition of total allowable catch (TAC) or minimum conservation reference size (MCRS).

Since the implementation of the CFP of the European Union (EU) in January 1983, the European Commission has repeatedly addressed the issue of discards (EC 1998; EC 2002*;* EC 2007*;* EC 2009). The LO was introduced in 2013 with implementation from 2015 (EC 2013); since then, de minimis exemptions have been established to this regulation for a high number of species and ICES regions, including the Mediterranean and the ICES areas VIII and IX, (EU 2018a*;* EU 2018b). Although under certain conditions it allows fishers to continue to discard species that would otherwise be subject to the LO, this will not prevent its full implementation in the coming years (Damalas 2015).

It is well documented that UWC, the reason for discarding, occur as a result of fishers’ choices at various stages of the fishing process (i.e., fishing strategies and tactics), and thus discarding patterns vary widely across space, time, fisheries, gears, and species (Adams et al. 2019*;* Chagaris et al. 2019*;* Nelson and Burnside 2019*;* Paradinas et al. 2016*;* Pennino et al. 2017*;* Rochet et al. 2014). It has been repeatedly demonstrated that discard patterns are motivated by the combination, in time and space, of two main components: (1) the distribution of density patches of potential UWC-prone species and (2) the distribution of the fishing effort. For example, density patches of potential UWC could be nursery areas of demersal fish, spawning aggregations of pelagic species or the occurrence of endangered species. These patches are characterized by spatial/temporal dynamics according to the life histories of the different species, the characteristics of the environment, and the distribution of the fishing effort (with particular reference to bottom otter trawling), which is the result of complex socio-economic drivers and interactions among fleets (Preciado et al. 2019*;* Russo et al. 2014). The inclusion of discards information into a spatial management perspective is in line with management procedures such as the common framework for Maritime Spatial Planning in Europe (https://ec.europa.eu/maritimeaffairs/policy/maritime_spatial_planning_en), representing a potentially ideal and effective approach to contribute to discard mitigation (Maina et al. 2018), and should be incorporated in discard management plans. The cross-analysis of fishing effort patterns and fish distribution can support the identification of spatial-temporal hot-spots (Maina et al. 2016), defined as areas and periods, in which the fishing pressure should be reduced to limit the amount of discards and the corresponding impact on the total biomass, species composition and diversity of the entire fish community determined by the discard/by-catch process (Eliasen and Bichel 2016*;* Pointin et al. 2018). This approach is also versatile because it can be tailored to each fishery or even species within a fishery.

According to this rationale, one of the key actions within the H2020 project MINOUW (“Science, Technology and Society Initiative to Minimise Unwanted Catches in European Fisheries”—http://minouw-project.eu/) consisted in assessing the spatial distribution of fishing effort in relation to areas of potential high discards. This was carried out in selected case studies by exploring remote vessel tracking data (VMS—Vessel Monitoring System/AIS—Automatic Information System) in combination with nursery and/or sensitive habitat maps. Here, we present the development approach and the design choices we employed in the context of the MINOUW program, applying the following workflow, data preparation and dissemination, to a total of seven Case Studies within the European waters (http://minouw-project.eu/case-studies-new/). As the purpose of this application is to demonstrate its potential to provide a framework for analysis, and also due to confidentiality purposes regarding these data, hereafter, a fictional case study near the Palmyra Atoll will be used as a reference example. The MinouwApp is open source and freely available with the fictional case study preloaded as a blueprint of the input requirements and formats. The complete code and the sample datasets can be downloaded from the Github repository (https://github.com/d-lorenz/minouwApp). The application can be loaded locally executing the source code, as is or eventually customized and adapted to new case studies, or it can be examined online with the live demo (https://minouw.shinyapps.io/minouwApp/).

## Development approach

**Fig. 1:**
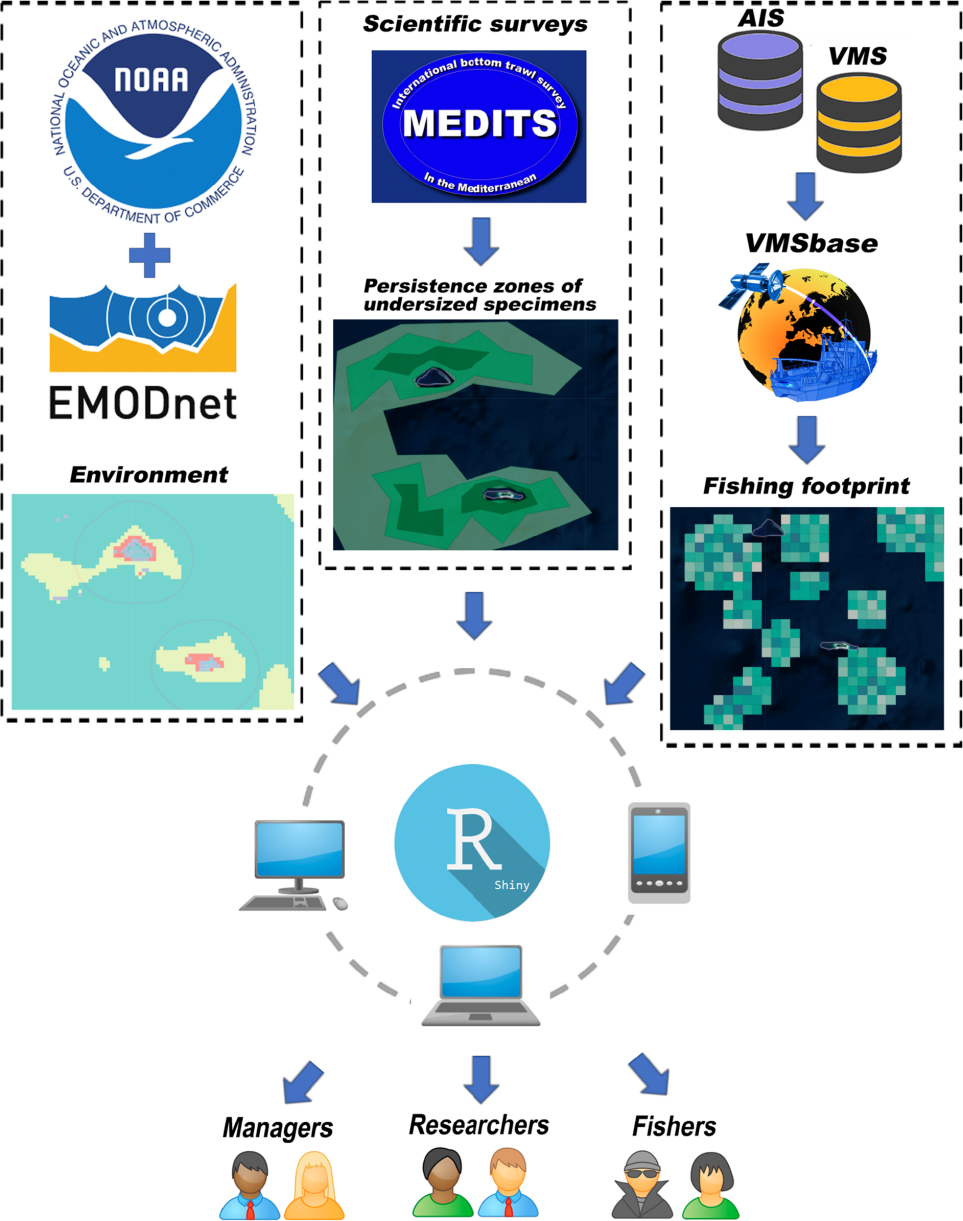
Representation of the main data sources integrated into the MinouwApp and of the main end users accessing the outputs. The logo of MEDITS, which is the main scientific survey carried out annually in the Mediterranean Sea to monitor the state of demersal fishery resources (Bertrand et al. 2002), is used here as a convenient example for whatever source of reliable information about the distribution of UWC

A web-based application was devised to allow for the visualization and the cross-analysis of different layers of metadata (Fig 1). The information to be used should aggregate data from multiple sources, comprising (1) maps of high-density patches of potential UWC (see “Unwanted catches” section), such as nursery areas of demersal fish, spawning aggregations of pelagic species or areas of distribution of endangered species. These maps can be obtained from routine trawl surveys (MEDITS: Mediterranean International Trawl Surveys, Bertrand et al. 2002; ICES-IBTS: (Atlantic) International Bottom Trawl Surveys, Niels et al. 2001), as well as national surveys carried out either in research and commercial vessels at the scope of the European Data Collection Framework (DCF) and, most importantly, fishery-dependent data obtained from vessel logbooks (2) habitat maps (“Habitat data” section), including sea bottom depth and substrate composition (e.g., EMODnet Seabed Habitats layers, https://www.emodnet-seabedhabitats.eu/); (3) fishing effort maps derived from AIS and VMS data (“Fishing footprint” section), the two main remote tracking devices currently used in fisheries science, providing high-resolution information on vessel activities.

The application described in this paper allows the inspection of the main components driving the patterns of discarding, explicitly illustrating their variability on the spatial and temporal dimensions. Namely, a series of overlap analyses to produce maps showing areas with a high probability to produce high discard rates of either commercial or by-catch species. The potential applications of this tool are related to the different end-users accessing it: (1) to fishers and related industry (e.g., ship-owners, fish processing, and marketing), which can use these maps to adapt their fishing strategies in compliance with the LO. Whether this can be provided in an almost real-time basis depends on whether georeferenced data on landings and discards from fishers logbooks are timely updated; (2) to managers, in support of informed management decisions such as the implementation of spatial closures and fisheries-restricted areas; (3) to researchers, by providing modelling approaches to estimate the potential effects of different management scenarios. Consequently, as all the stakeholders have access to the same spatially explicit information and share a common platform, they are therefore empowered by the opportunity to co-create possible management solutions.

From a technical point of view, the application described in this paper, called MinouwApp, is based on Shiny (Chang et al. 2015), an R package to build interactive web apps straight from R, which is the most widespread statistical environment in fisheries science (https://www.r-project.org/). Several publications of Shiny web applications, covering a wide range of scientific fields, were made available in recent years. The ones dedicated to spatial data and including GIS database include a web-based mapping application for precision farming (Jahanshiri and Shariff 2014) and a web-based screening model for climate risk to water supply systems in the northeastern USA (Whateley et al. 2015).

The MinouwApp integrates not only a series of geodatabases, independently obtained by different MINOUW teams of researchers with different methods and data sources, but also layers of spatial information coming from other sources, including open-access data and results from EU research projects.

### Design choices

**Fig. 2:**
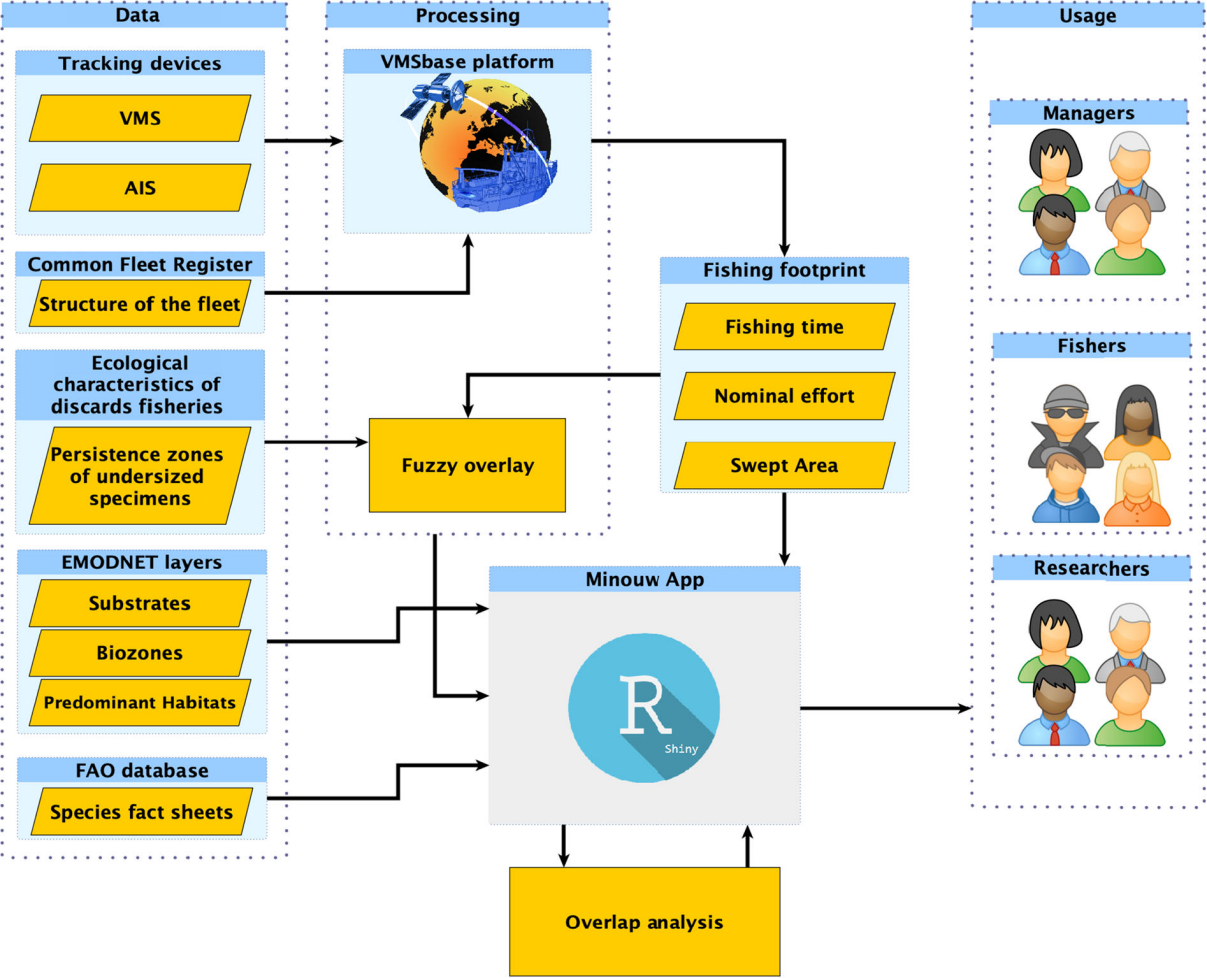
Schematic representation of data- and workflow regarding the preparation and deployment of each case study

The protocol of data analysis has been arranged, for each case study, in three subsequent steps: data collection, processing, and usage (Fig. 2). During the data collection step, all the datasets (detailed in the following sections) required for downstream processing were compiled and harmonized. The data processing step is responsible for the transformation of the raw data on the individual (vessel’s) fishing activity into an aggregated footprint of the fishing. This is a mandatory step for two different reasons: first, because both the desired output of the project and the customarily adopted methods of analysis involve the use of data aggregated at the fleet level; secondly, the individual fishing activity includes personal information, whose disclosure we chose to avoid for reasons of confidentiality. Finally, the processed datasets are properly wrapped into a single Shiny App package and loaded online on the ShinyApps.io cloud service to be made available to authorized users.

### Data

To conduct a complete set of analyses, and to fulfil all the required steps of the MinouwApp workflow, several sources of information are required, and the mandatory datasets are described in the following subsections.

#### Boundaries and spatio-temporal units of the area of study

The geographical boundaries of each case study are delimited by a shapefile composed of a polygon layer enclosing all the maritime surfaces to be assessed. A regular grid of rectangular cells, built on the extent of the former shapefile, is another essential object needed to define the spatial dimensions of the study. Each cell of the grid, associated with the corresponding C-Square string (Rees 2003), will become the basic spatial unit to which any further computed measure will be related.

The required dataset is temporally heterogeneous, and usually collected or organized at multiple temporal scales: survey data, which are the foundation for the UWC, are based on annual or seasonal surveys or fishing logbooks; VMS/AIS data are logged with a frequency ranging from minutes to hours but then aggregated at the monthly scale; the available output on the MinouwApp is aggregated quarterly and yearly for visualization purposes. The patterns of effort are the more dynamic effect accounted in the system, while the UWC maps are assumed to be more stable across seasons/year (with the necessary precautions based on individual species characteristics). Being the fuzzy relationship computed quarterly or yearly, the general guideline is to employ a coherent temporal range between the two time-series with a complete overlap between the two sets, since the fuzzy-product effort/UWC will be applied on the same time-window (each quarter or year).

#### Habitat data

The habitat data in this application is a collection of measured or modelled information to illustrate the bottom floor features of the area of study in terms of seabed characteristics and bathymetric profile. It is downloaded, as a shapefile with multiple attribute fields, from the Seabed Habitats portal of the EModNet project (http://www.emodnet.eu/). The raw seabed features are clipped with the case study boundary shapefile and only the “Biozone,” “Substrate,” “EUNIS classification,” and “MSFD habitats” fields are kept. The second dataset, i.e., the bathymetric profile, is obtained from the ETOPO1 database hosted on the NOAA website. The “marmap” R package (Pante and Simon-Bouhet 2013) which is dedicated to gathering and transforming bathymetric data was used to download and convert the bathymetric dataset to the raster layer format with the maximum available resolution of one degree.

#### Fleet register

The structural characteristics of any fishing vessels actively engaged in the case study area should be gathered from a dedicated repository. Within the MINOUW project, the European fleet register online database available on the Fleet Register On the Net (FRONT, http://ec.europa.eu/fisheries/fleet/index.cfm) website was used. Assuming that this source of information is available, the final product is arranged as a table including the fields about the vessel identification number, length over all (LOA), horsepower, and main and secondary fishing gear.

#### Fishing footprint

For each case study, the fleet was defined in terms of fishing vessels composition and characteristics. The fishing footprint was estimated at a monthly scale, for the period 2012–2016, using all the VMS/AIS available data, following the workflow of the VMSbase platform (Russo et al. 2014*;* Russo et al. 2016). The fishing footprint was then coupled with habitat maps to identify the hot spots of discards production, based on two different overlap approaches (see details in “Fleet register,” Fuzzy overlay,” and “Overlap analysis” sections).

The raw VMS/AIS data of the fishing fleet involved in the studied fishery system is processed with the vmsbase R package (https://cran.r-project.org/web/packages/vmsbase/index.html) and archived as SQLite databases. Subsequently, the standardized fishing positions are used to prepare the aggregated effort metrics to be loaded on the MinouwApp. The fishing effort metrics computed are “Fishing Hours,” “Fishing Days,” “Nominal Effort,” and “Swept Area.” In more detail:

1. The Fishing duration was computed as the product of the number of fishing points (from VMS/AIS databases) by their time interval (10 min);
2. The Nominal Effort was computed as the product of the fishing days (FD) and the engine power (PW in kW) of each vessel. The total FD of each vessel for each month was computed using the R package fecR (Scott et al. 2017). For example, in case that a single gear is in use during a fishing trip, it was allocated 1 fishing day for each date with at least one point classified as “fishing activity” (e.g., one or more tows);
3. The Swept Area (SWA) was computed, for the case studies involving towed gears (trawling) according to Eigaard et al. *(*2016*)* *and* Russo et al. *(*2017*)* as follows:
  $$ SWA= OFS\times TL $$

where *OFS* is the trawl horizontal opening, i.e., distance between trawl wing tips, and *TL* is the length of the haul (in meters). While *TL* can be directly computed from interpolated pings corresponding to Fishing Points, *OFS* was estimated, for each fishing vessel length (LOA), using the following formula:

$$ OFS=a\times LOA+b $$

The *a* and *b* parameters (respectively, slope and intercept of the linear link function) were selected, for each case studies, from Table 4 of Eigaard et al. *(*2016*)*. The computation of the Swept Area was not carried out for the Case Studies involving active gears (i.e., Purse Seine).

#### Unwanted catches

The last input demanded is the shapefiles of the UWC and the FAO factsheets for the studied species. The UWC shapefiles, created by the end users from georeferenced survey data, consist of a collection of geospatial layers depicting the potential zones of undersized organisms resulting from the computation of the Persistence Index (PI, Fiorentino et al. 2003*)*. The UWC shapefiles can be prepared following the methodology proposed in Colloca et al. (2009)*. Briefly,* a time series of juvenile densities is estimated from survey data with Bayesian kriging and afterwards, a geostatistical aggregation accounts for the density hot-spots. The resulting PI measures the number of times an area is classified as a hot spot, for organisms under the minimum landing size, with reference to the total number of years in the time-series. Specifically, higher PI values mean higher hot spot occurrence throughout the time series. Otherwise, when the PI value is zero, a hot spot has never been observed. We employed single-species maps of areas of high juvenile densisty; however, other hot-spot identification criteria (i.e., endangered species or sensitive habitats) can be employed with the condition of being provided as a polygon shapefile of areas with potential UWC.

### Processing

The processing phase (Fig. 2) comprises two blocks of analyses carried out outside the MinouwApp. Each block is based on a published procedure, which was already applied and tested outside the MINOUW project, namely, the VMS/AIS processing and the Fuzzy Overlay technique. The integrated analysis of VMS and AIS data with the VMSbase platform is extensively documented in Russo et al. (2014) and Russo et al. (2016). The Fuzzy Overlay combines both fishing effort and UWC by taking into consideration two types of layers: (1) the spatiotemporal distribution of fishing effort and (2) the spatial distribution of UWC by each species under investigation. The use of the fuzzy logic technique has started gaining recognition in fisheries science in the last decades (e.g., Stelzenmuller et al. 2010*;* Kavadas et al. 2015). Fuzzy logic represents an extension of the classic binary logic, with the possibility of defining sets without clear boundaries of elements belonging to a given set (Malczewki 1999*;* Biacino and Gerla 2002). A Fuzzy Linear membership function was used to transform the values of each type of layer to a 0 to 1 possibility continuous scale. Linear transformations are commonly used in the fuzzification of deterministic criteria (Fisher 2000). The Fuzzy Linear transformation was applied between the minimum (min) and maximum (max) of the values distribution and is estimated by the formula:

$$ {\displaystyle \begin{array}{c}\mu (x)=0\  if\ x<\min, \mu (x)=1\  if\ x>\max, \\ {}\mathrm{otherwise}\ \mu (x)=\frac{\left(x-\min \right)}{\left(\max -\min \right)}\end{array}} $$

where *x* is the value in each cell (planning unit).

The spatial distribution of the combination of both fishing effort and UWC was derived as the fuzzy overlay of the two previously Fuzzy-transformed types of layers. Generally, there are several techniques used in a fuzzy overlay analysis, for investigating the relationships and quantifying the interactions. The combination approach used in this work was the Fuzzy Product (Zimmermann and Zysno 1980). The Fuzzy Product overlay type will, for each cell, multiply each of the fuzzy values for all the input criteria. Values close to 1 indicate areas with an elevated likelihood of intense fishing effort and UWC (at the same time). It should be noted that despite the apparent similarity between the concepts of “fuzzy overlay” and “overlap analysis,” their functioning and purpose are different and complementary. While the overlap analysis is a GIS routine to select specific areas (according to the effort and UWC thresholds) and then merge continuous patches of cells, the fuzzy overlay performs a fuzzy transformation (it standardises the observed distributions, of both effort and unwanted catches, to be between zero and one) and applies a fuzzy product, to provide a combined representation of both the distributions.

### Usage and end-users

**Fig. 3:**
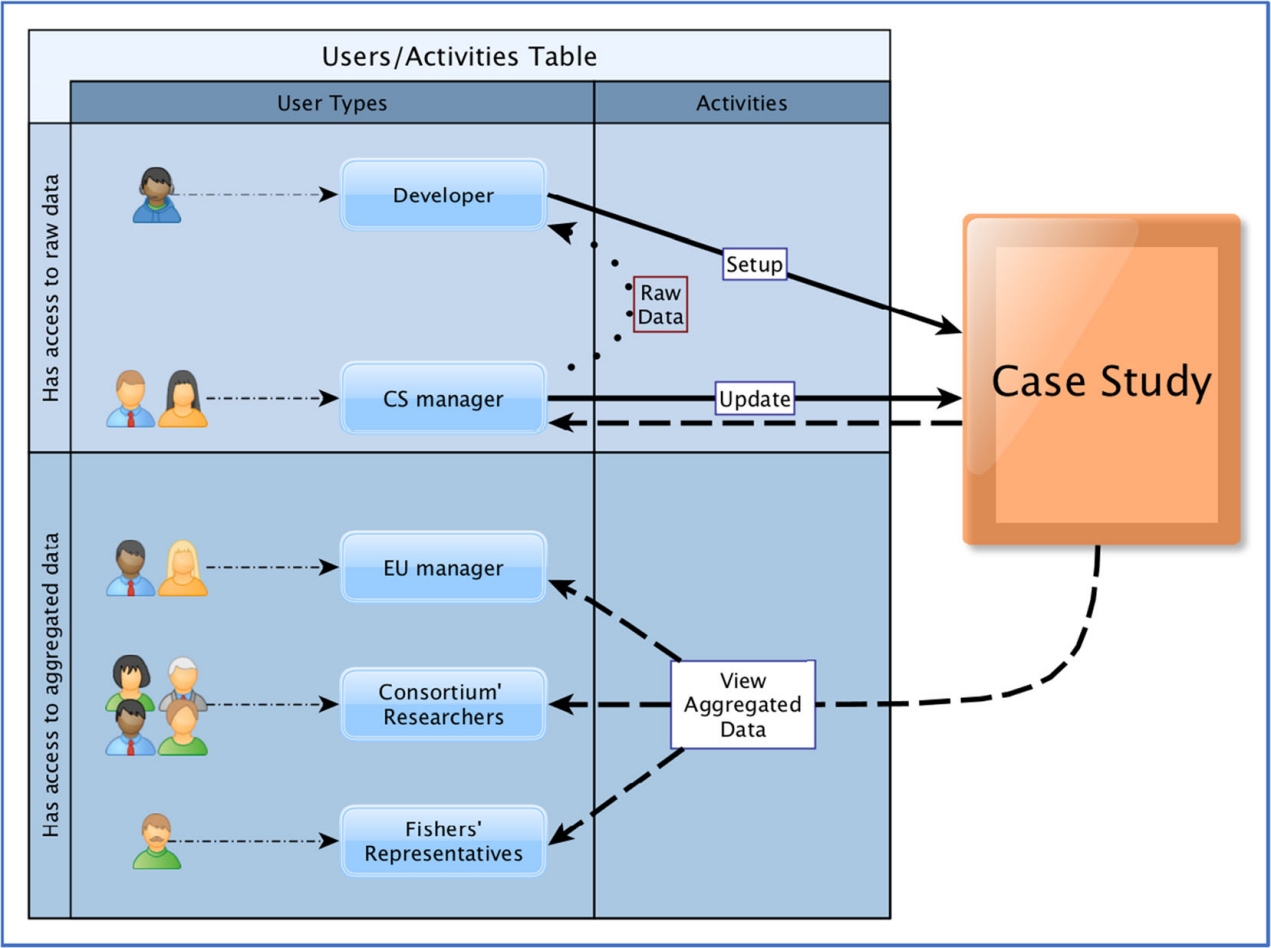
Case study (CS) access rights schema and activity routes by user type. The developer and the CS managers oversee the setup and update of the CS data, while the other group of users is allowed access to the aggregated data only

The application of the MinouwApp to each case study considered within the MINOUW project involved the developer of the MinouwApp and at least one researcher as “case study expert” (Figs. 1, 2, and 3). Raw data, including VMS positions (which are confidential), and AIS positions, were exchanged only between the developer team and the case study expert in order to avoid the inadvertent leak of sensitive data. At the end of the processing phase, the aggregated pattern of the fishing footprint was obtained from VMS and AIS data and uploaded into the MinouwApp.

With reference to the MinouwApp usage, it is possible to list three main groups of end users: managers, researchers, and fishers, whose interaction capabilities with the dataset are relegated to the aggregated data (since it is the sole information that is actually uploaded online). Furthermore, every single user must be individually authorized to access a specific case study.

## Implementation and panel structure

The MinouwApp is designed as a Shiny Dashboard. The dashboard consists of two main objects, a vertical sidebar on the left, which functions as a clickable menu, and the body of the page on the right, which shows the different interactive information related to the case study. The user can then navigate between the different information panels clicking on the corresponding menu buttons. In practice, the following options are available: “Overview,” “Effort Data,” “Unwanted Catches,” “Fuzzy Overlay,” “Overlap Analysis,” “Wordbook,” and “References & Credits.”

### Overview

**Fig. 4:**
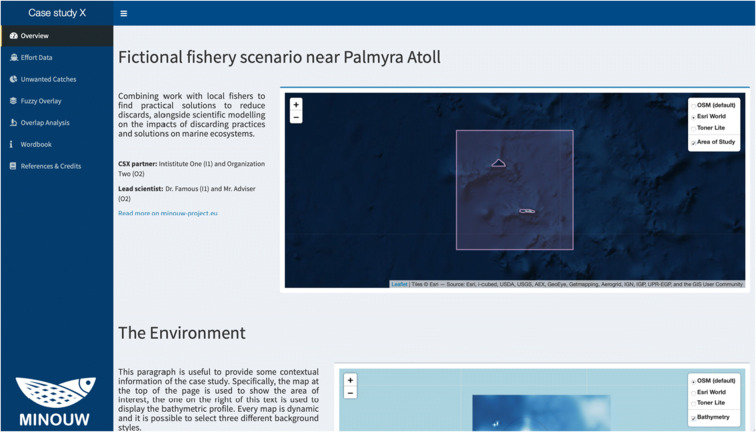
Screenshot of the Overview panel, with an example from the case study of bottom trawl fisheries in the fictional case study near Palmyra Atoll

The “Overview” page (Fig. 4) is designed to supply the user with the basic information about the specific case study under examination, being divided into three subsections. The first corresponds to a brief description with scope and objectives, names of the scientific officer and partners, and an interactive map focused on the area of interest along with a highlight of the actual maritime zone that defines the spatial limits of the case study. The second contains a more detailed description of the fishing system under examination and two maps, one with the bathymetric profile of the area and the other with three different levels of information about the type of substrate and seabed category. The last subsection is used to report the relevant information about the fishing fleet and includes a text summary and a graphical representation of the fleet characteristics.

### Effort data

**Fig. 5:**
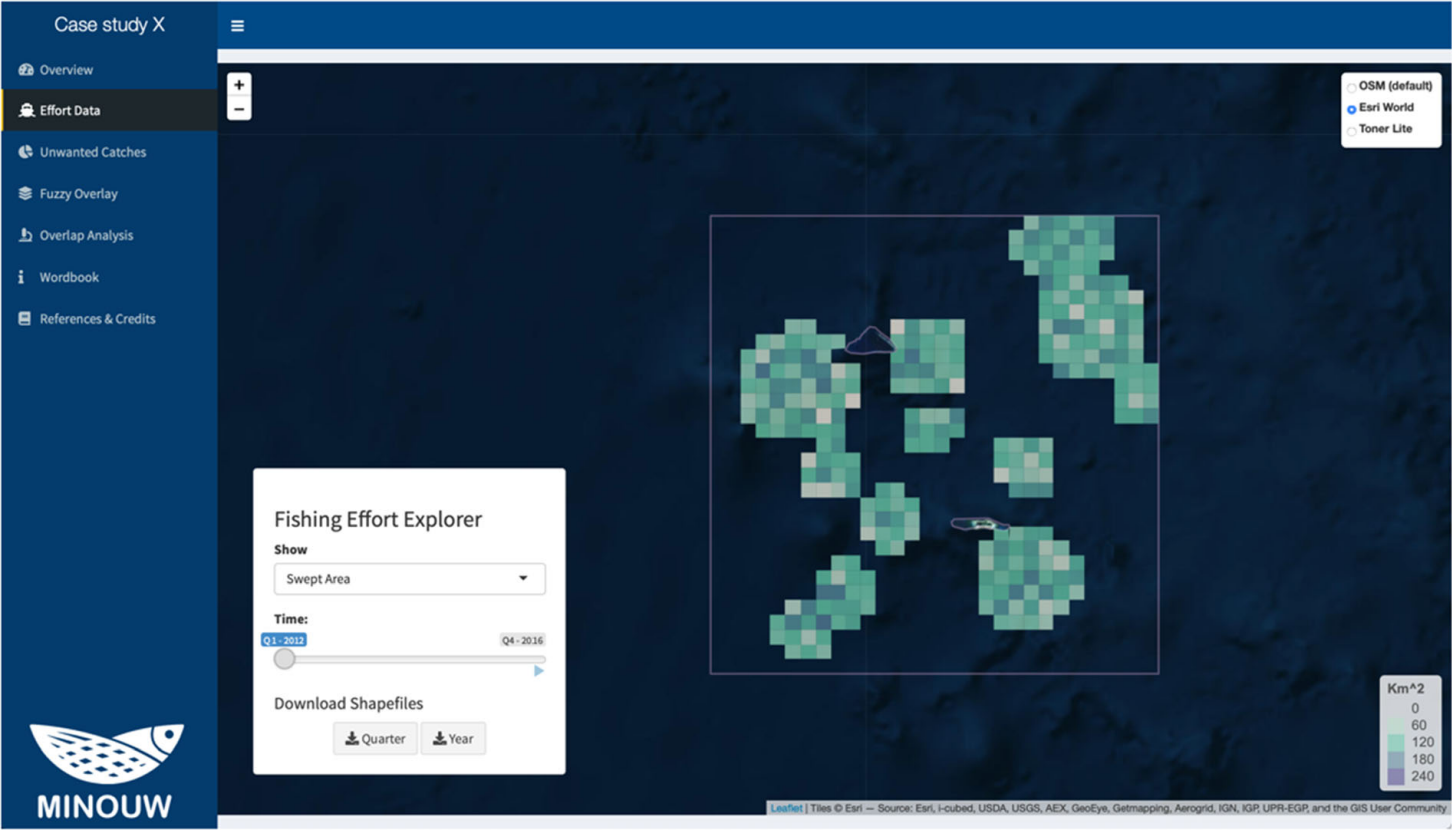
Screenshot of the Effort data panel, with an example from the case study of bottom trawl fisheries in the fictional case study near Palmyra Atoll. In this example, the fishing effort is represented as the swept area (km^2^)

The “Effort Data” page (Fig. 5) provides a large interactive map to explore the spatial dynamics of the fleet through four different fishing effort metrics selectable by the user between Fishing Hours, Fishing Days, Nominal Effort, or Swept Area. The map shows the selected distribution as various shades of a colour ramp, darker blue and lighter green colours denoting higher and lower values, respectively. While the basic unit of the temporal resolution showed is the quarter, the user can download both the quarter and the yearly temporal aggregation. It is possible to change the time frame by moving the slider to select a different quarter or automatically animate the map clicking on the play button.

### Unwanted catches

**Fig. 6:**
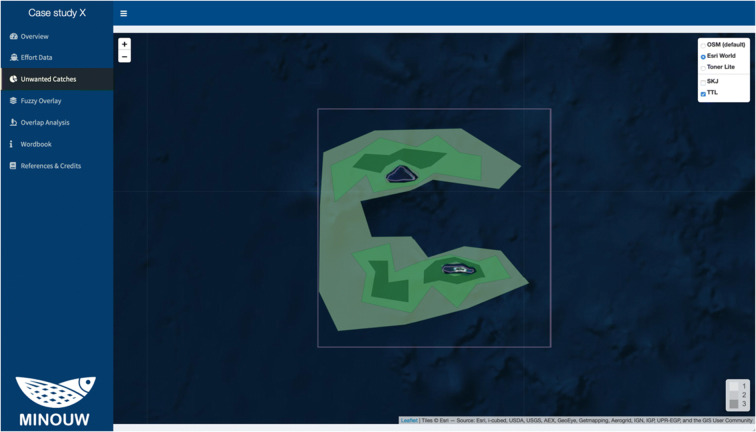
Screenshot of the Unwanted catches panel, with an example from the case study of bottom trawl fisheries in the fictional case study near Palmyra Atoll. In this example, the different polygons are coloured with respect to the Persistence Index scale in the bottom right side of the map

The “Unwanted Catches” page (Fig. 6) allows visualizing the UWC Map, which represents graphically the different areas where a number of catches below the minimum landing size (MLS) have occurred within the area of study. The color-coded shading represents the level of occurrence of UWC (Persistence Index), light colours for low persistence (the area is classified as hot-spot only in 1 year of the time-series) and darker colours for higher persistence (the area is classified as hot-spot every, or at least more than one, year of the time-series). The user can select and deselect the different species in display by clicking on the species three-code name on the top right menu of the map.

### Fuzzy overlay

**Fig. 7:**
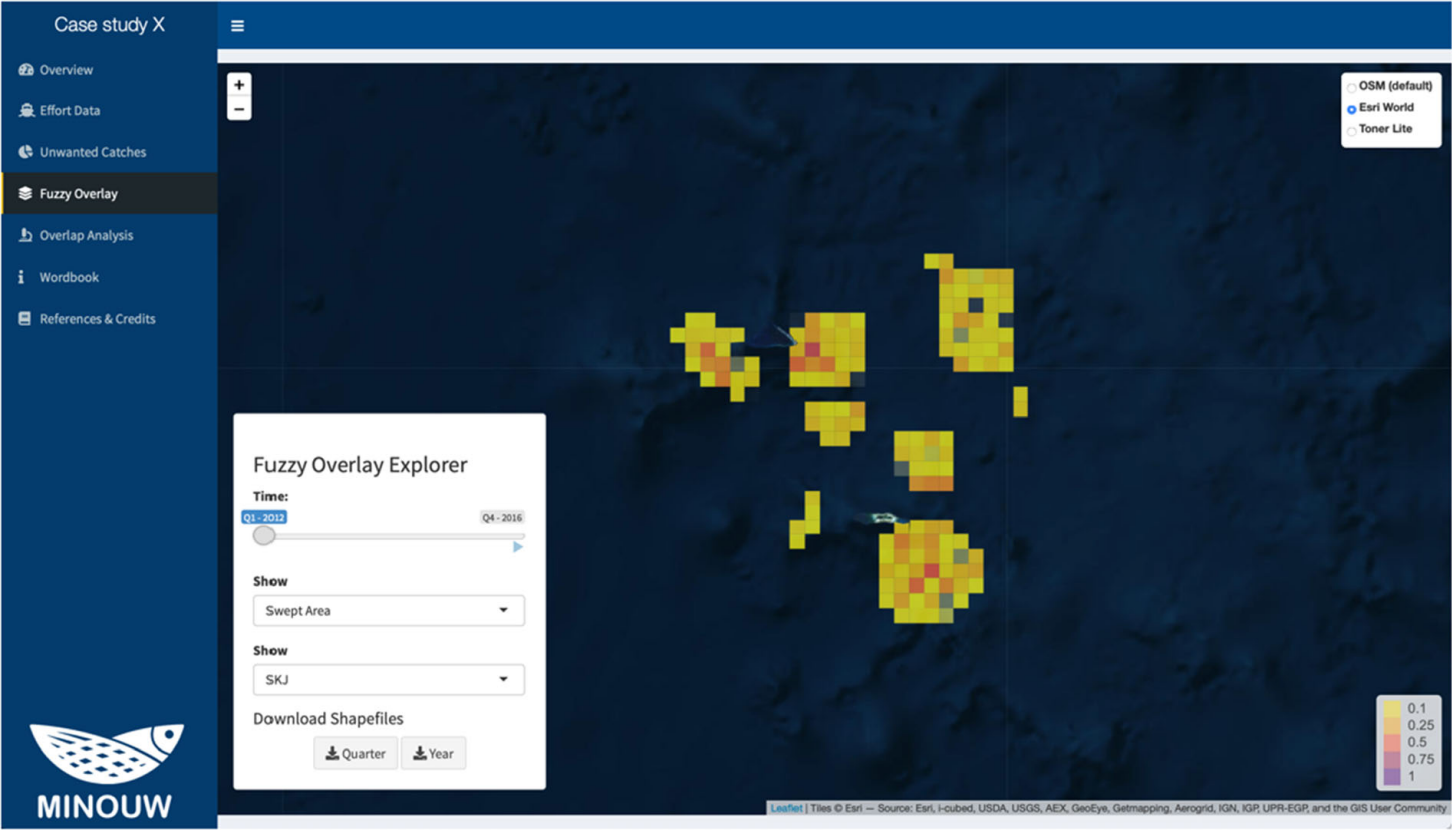
Screenshot of the Fuzzy overlay panel, with an example from the case study of bottom trawl fisheries in the fictional case study near Palmyra Atoll. In this example, the different cells are coloured with respect to the (0–1) index scale in the bottom right side of the map

The “Fuzzy Overlay” page (Fig. 7) shows an interactive map with the results of the fuzzy overlay procedure. The interface displays the fuzzy product, on a single time frame, corresponding to the selected fishing effort metric and a species. The Fuzzy Overlay index is on a scale between 0 and, with zero meaning “no overlap” and one “total overlap.” The time frame can be chosen by the user using the slider on the fuzzy overlay explorer or automatically animated clicking the play button. It is possible to download the data on both quarterly and yearly aggregated time frames.

### Overlap analysis

**Fig. 8:**
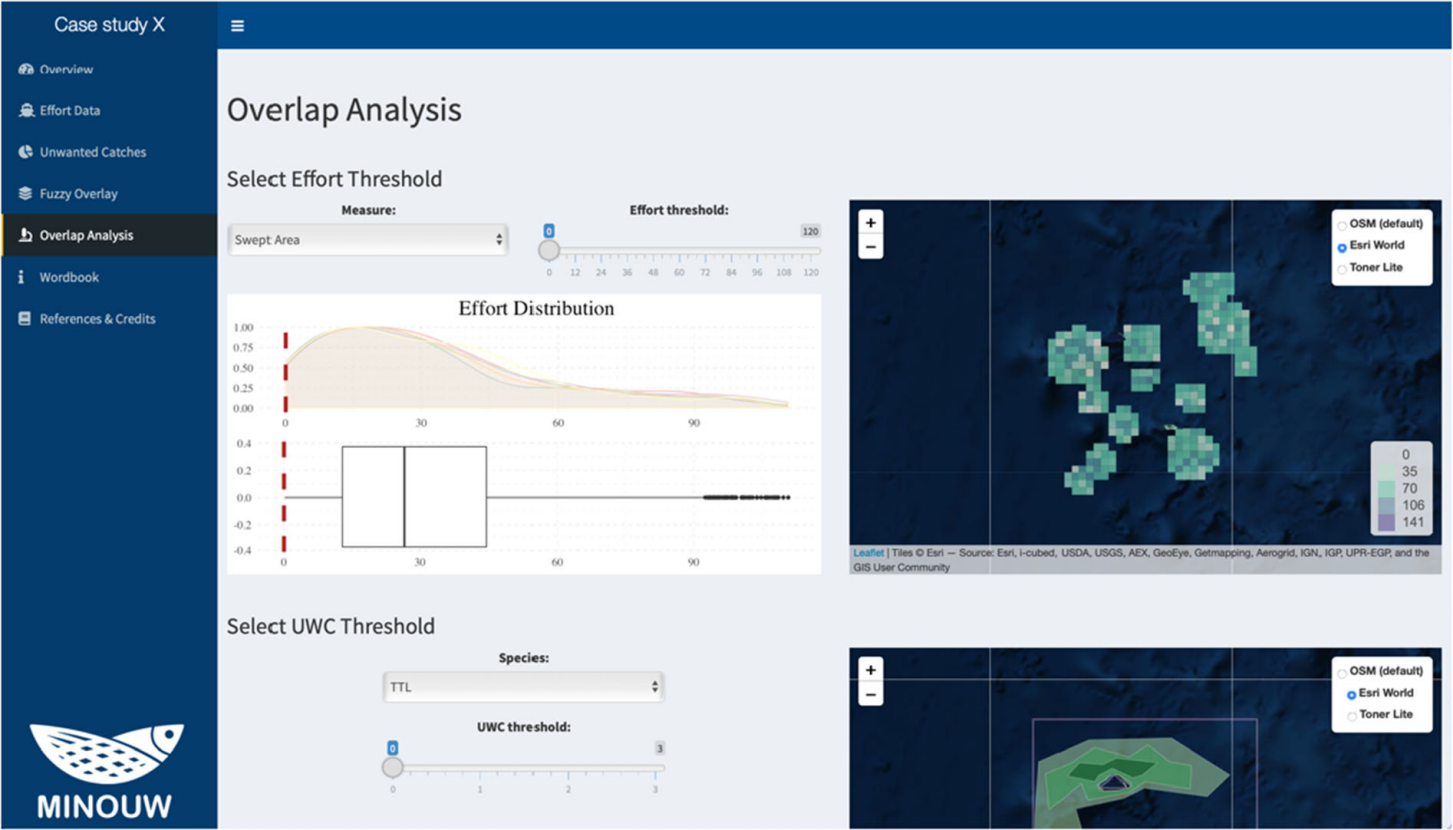
Screenshot of the Overlap analysis panel, with an example from the case study of bottom trawl fisheries in the fictional case study near the Palmyra Atoll

Although the detailed cross-analysis of fishing effort and discards patterns is beyond the scope of this application, the MinouwApp interface allows producing some basic overlap analyses (Fig. 8). Namely, the user can:

- Select a measure of the fishing effort;
- Define a threshold range for the fishing effort measure;
- Select a species for the UWC layer;
- Define a threshold range for the UWC layer;
- Perform an overlay analysis between the area of UWC and the pattern of the fishing effort.

The MinouwApp will return, as a visual output, the union of the contiguous cells meeting the selected thresholds and a table of the main related statistics. Clicking on the button at the bottom of the page, the resulting spatial selection can be also downloaded as a shapefile.

### Wordbook and references

**Fig. 9:**
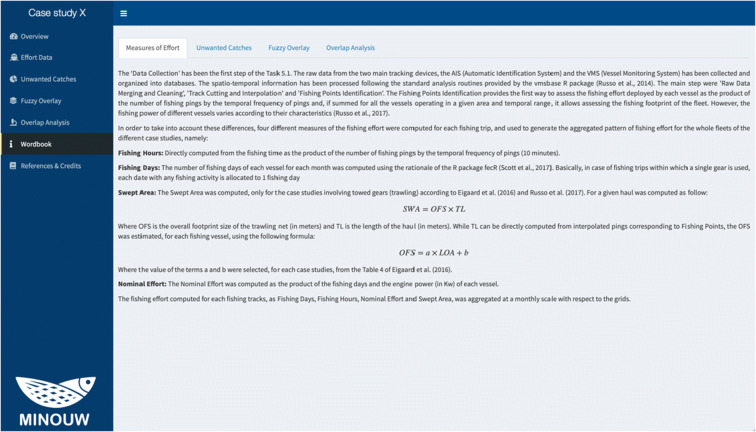
Screenshot of the Workbook panel, with the explanation of the procedure for the computation of the different metrics applied to quantify the fishing footprint

The last two pages of the MinouwApp, “Wordbook” and “References & Credits,” report a collection of the basic knowledge relevant to the understanding of the Minouw Project, the MinouwApp, and the specific case study. Four panels are presented in the “Wordbook” page (Fig. 9): Measure of Effort, Unwanted Catches, Fuzzy Overlay, and Overlap Analysis. In each one of these panels, a brief explanation is provided on the underlying procedure employed to perform the analysis along with a short definition of the terms used. In the Unwanted catches panel, there are other subsections showing the FAO fact sheet of the species included in the case study.

**Fig. 10:**
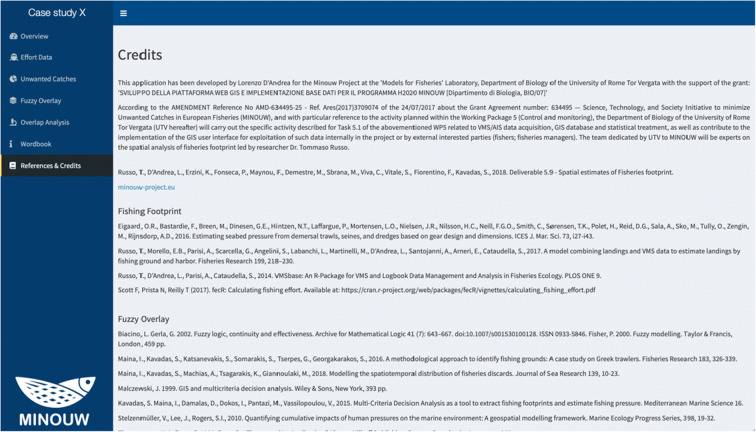
Screenshot of the Credits panel

The “Credits” page (Fig. 10), other than the acknowledgement of authorship and funding for the Minouw project, is a collection of the relevant literature to each section of the MinouwApp.

## Discussions and conclusions

The estimation of the fisheries footprint and of the species’ geographical distribution represent two fundamental steps for a spatially based resource management system designed to achieve the sustainability of human activities in the marine space. Sustainability is, in turn, a multidisciplinary concept that involves environmental, socio-economic, and jurisdictional aspects. This means that minimizing human impacts at sea require the involvement of different actors, including scientists, managers, fishers, and other stakeholders, along with, considerable diversification of competences. Within the specific thematic of discard reduction, but also in the general context of fisheries management, there is an urgent need to seek practical solutions to address the complex issue of balancing human needs with sustainable exploitation of marine ecosystems. The current situation is additionally complicated by both the inherent difficulties deriving from the interaction of a community with mixed know-how participating in the decision process and the essential non-triviality of the methodological approaches to be formulated and proposed to relieve the pressure on the biological resources. Another key aspect to highlight, for improved comprehension of the behavioural dynamics of a fishery system, is the emphasis to be recommended about the explicit consideration and the methodological inclusion of the spatial dimension and of the advantages coming from its leverage.

In consideration of the stated assumptions, the development, distribution, and update of an operational platform are critical for (1) a deeper insight on fishing effort, bycatch, discards, and stock spatial distribution and their relationship; (2) facilitating data analysis and presentation of crucial findings of ongoing research; (3) identifying hot-spot areas for priority management intervention; and (4) providing managers with an operational tool in support of decision. The MinouwApp represents one of the first attempts towards this, through the design of a technological and methodological framework for the inclusion and harmonization of all the mentioned objectives. Furthermore, the purpose of this application is to equip the involved parties with a convenient “control centre” for an express acknowledgment of the fishery’s structure (fished populations and fleet interaction), able to provide a straightforward appraisal of the current circumstances of resource exploitation on the spatiotemporal dimensions. Specifically, the MinouwApp implements:

- The main dashboard to summarize the main typical feature of the fishing fleet together with the details of the environment in which it operates;
- Three main GIS mapping tools to inspect, first, the dynamics of the fishing fleet on the basis of different measures of effort; second, the geographical distribution of the studied species and the major areas of potentially unwanted catches; third, the combination of the previous two descriptive informational layers for the integrated evaluation of fish and fishers spatial concentrations;
- An adequate interface to perform the fundamental operations of selection, overlap, and union of the geographical areas of principal interest;
- A simple, yet precise, glossary page for a succinct explanation on both the terminology used and the essential biological aspects of the species in study.

The description of the MinouwApp and the example of its application to the provided fictional case study demonstrate the feasibility in coordinating large amounts of data from different sources. These include data collected within scientific surveys, generated through remote tracking devices onboard fishing vessels as well as open-access data from different origins, into a visual interface allowing to interactively select temporal ranges and areas of interest and return maps (with related statistics) that can both support management strategies, such as the development of regional Discard Plans, and drive fishers in adapting their behaviour to meet legislation requirements. In addition, the MinouwApp can be further tailored to support additional aspects of fisheries spatial management from the supervision of marine management areas to the designation of fisheries restricted areas or the identification of vulnerable marine ecosystems (VME), in line with CFP and MSFD objectives.

This application constitutes a framework for the inspection of discarding patterns and their main drivers. It represents a novel, participative approach in fisheries science (i.e., as a powerful Research & Innovation tool within a scientific support system, https://ec.europa.eu/info/research-and-innovation/strategy_en), by offering an opportunity for managers, fishermen, scientists, and the public to be involved at a regional level in designing frameworks that improve global understanding of the ocean and incentivize long-term fisheries sustainability. Whether this information can be used in support of governance depends on whether short-term decisions are allowed, by providing georeferenced data on landings and discards in an almost real-time basis. Lastly, fisheries struggle to balance the exploitation of a publicly held resource (i.e., the fish) while maintaining the confidentiality and intellectual property of the fishermen. In fact, the general policy status tries to accommodate the different interests between publicly available knowledge, intellectual property, and confidentiality while avoiding hindering the research and the potential impairing of our capabilities to foresee, prevent, and treat environmental issues. Thus, we strived to provide all the necessary aspects of the dataflow for the MinouwApp as a publicly available platform (on the GitHub repository: https://github.com/d-lorenz/minouwApp) to be employed on future case studies.

## Abbreviations

AIS: automatic identification system
CFP: common fisheries policy
EMODNET: European Marine Observation and Data Network
FAO: Food and Agriculture Organization
FD: fishing days
FRONT: Fleet Register On the Net
GIS: Geographic Information System
LO: landing obligation
LOA: length over all
MEDITS: MEDiterranean International Trawl Surveys
MSFD: Marine Strategy Framework Directive
NOAA: National Oceanic and Atmospheric Administration
OFS: overall footprint size
PI: persistence index
PW: engine power
SWA: SWept Area
TL: total haul length
UWC: unwanted catches
VME: vulnerable marine ecosystems
VMS: vessel monitoring system
